# Effect of transcutaneous auricular vagus nerve stimulation on stress regulation: an EEG and questionnaire study

**DOI:** 10.3389/fdgth.2025.1593614

**Published:** 2025-09-01

**Authors:** Woo-Jin Kim, Yun-Sung Lee, Ki Hwan Hong, Hyuk Choi, Jae-Jun Song, Han-Jeong Hwang

**Affiliations:** ^1^ Department of Electronics and Information Engineering, Korea University, Sejong, Republic of Korea; ^2^ Interdisciplinary Graduate Program for Artificial Intelligence Smart Convergence Technology, Korea University, Sejong, Republic of Korea; ^3^ Neurive Co., Ltd., Gimhae, Republic of Korea; ^4^ Department of Medical Sciences, Graduate School of Medicine, Korea University, Seoul, Republic of Korea; ^5^ Department of Otorhinolaryngology-Head and Neck Surgery, Korea University Guro Hospital, Seoul, Republic of Korea

**Keywords:** transauricular vagus nerve stimulation (taVNS), stress, electroencephalography (EEG), power spectral density (PSD), brain network

## Abstract

**Introduction:**

Transcutaneous auricular vagus nerve stimulation (taVNS) has demonstrated efficacy in alleviating stress-related symptoms, yet its underlying neurophysiological mechanisms remain incompletely understood. In this study, we aimed to elucidate the effects of taVNS on stress regulation by employing self-report surveys and electroencephalography (EEG) measures.

**Methods:**

Participants performed mental arithmetic (MA) and 2-back tasks to induce stress, receiving either taVNS or sham stimulation in a randomized, crossover design. Resting-state EEG was recorded at baseline and immediately after each stress-inducing task—thereby capturing persisting taVNS-induced changes in neural activity—and subjective stress levels were assessed immediately before and after stimulation.

**Results:**

The survey results showed a significant increase in perceived stress following the tasks in the sham condition, whereas stress levels in the taVNS condition did not significantly change. Power spectral density (PSD) analyses revealed that theta and alpha band power in the frontal region significantly decreased only in the sham condition, suggesting elevated stress. Furthermore, whole-brain network analysis indicated a significant reduction in theta band path length in the taVNS condition following both tasks, pointing to enhanced global network efficiency that may help prevent stress escalation.

**Discussion:**

By demonstrating distinctive EEG and behavioral differences between taVNS and sham stimulation, this study provides a neurophysiological basis for taVNS as a non-invasive intervention for stress regulation. Future research involving a more diverse participant pool and optimization of taVNS parameters will further clarify its therapeutic potential.

## 1 Introduction

Stress occurs continuously in daily life and can negatively impact mental and physical health. When stress is perceived, the autonomic nervous system (ANS) and the Locus coeruleus (LC) become activated, potentially disrupting homeostasis and leading to various physiological and psychological issues (1, 2). For example, stress increases the risk of developing depression (3) and can contribute to disorders such as post-traumatic stress disorder (PTSD) when individuals are exposed to severe stress (4). To treat disorders induced by stress, psychological counseling and pharmacological treatment methods are commonly used (5). However, due to factors such as drug resistance, these conventional treatment methods have shown meaningful efficacy in only about two-thirds of patients with stress-related disorders (6, 7).

Vagus nerve stimulation (VNS) has been proposed as an alternative therapeutic approach for alleviating symptoms in patients for whom standard treatments are ineffective, such as in cases involving drug resistance (8). VNS is a stimulation technique utilizing low-level electrical currents to stimulate the vagus nerve and has been shown to modulate both the ANS and LC, thereby influencing stress-related neural circuits (9, 10). VNS is a stimulation technique utilizing low-level electrical currents to stimulate the vagus nerve, and it has been applied effectively in treating disorders including depression (11, 12), epilepsy (13, 14), and stroke (15, 16). Additionally, VNS has shown efficacy in patients who respond poorly to conventional treatments (17–19). Nevertheless, VNS is generally used selectively in severe cases, as it requires surgical implantation of stimulation devices, such as electrodes and generators.

Recently, transauricular VNS (taVNS) has been proposed as a non-surgical alternative to VNS. taVNS does not require invasive procedures and has been shown to effectively alleviate symptoms associated with stress-related disorders, including depression and PTSD, similarly to conventional VNS (20–22). However, because neural mechanism underlying the beneficial effects of taVNS remain unclear, numerous researchers have explored its effects on brain activity using various neuroimaging techniques (23–25).

Among neuroimaging methods, electroencephalography (EEG) is extensively used in taVNS studies due to portability and high temporal resolution (25–29). For example, Ricci et al. (30) reported increased delta power during 60 min of taVNS in a resting state (30). Lee et al. (31) confirmed the neuromodulatory effects of taVNS using resting-state EEG and proposed optimal stimulation durations for achieving effective outcomes (31). In addition, EEG is frequently utilized to study changes in brain activity according to various mental states, particularly stress. Wen et al. (32) reported that theta and alpha frequency powers in the frontal region were inversely correlated with stress levels (32). Interestingly, theta and alpha band activity has also been shown to vary depending on sleep stages, with anti-correlation in REM sleep (associated with higher stress) and positive correlation in deep sleep (associated with lower stress), suggesting their relevance to the brain's stress regulation mechanisms during both wake and sleep states (33). Yan et al. reported that beta frequency power in the frontal region was higher under stress conditions compared to non-stress conditions (34). Additionally, Shim et al. reported that PTSD patients showed reduced theta network indices compared to healthy controls (35). Dimitrakopoulos et al. (36) found decreased network indices under high workload conditions related to low workload conditions (36).

Although some studies have investigated taVNS-related changes in brain activity using EEG or examined EEG changes associated with different stress levels, there is still a relative lack of research exploring the specific neural mechanisms and effects of taVNS across varying stress levels. In particular, most previous studies assessing the impact of taVNS on stress have relied on electrocardiogram (ECG) measurements (37–39), leading to an underrepresentation of EEG-based approaches in the literature. Furthermore, given the correlations between stress and brain activity, it is essential to investigate both the effects and the underlying neural mechanisms of taVNS on stress using EEG. Taken together, these findings suggest that EEG may be a more suitable modality for examining how taVNS modulates stress and brain activity.

In this study, we aimed to investigate the effects of taVNS on stress using both survey and EEG data. To achieve this, participants underwent either taVNS or sham stimulation during stress-inducing tasks. Resting-state EEG was recorded before and after each task, and self-report surveys were administered both before and after stimulation. Subsequently, power spectral density (PSD) and network indices were analyzed across three frequency bands—theta (4–7 Hz), alpha (8–13 Hz), and beta (14–30 Hz)—which have been consistently associated with stress in previous studies. In contrast, delta and gamma bands were not included in the analysis due to their inconsistent or limited relevance to stress-related EEG changes reported in the literature (40, 41).

## 2 Materials and methods

### 2.1 Participants

Fourteen healthy individuals initially participated in this study. However, due to technical issues during data collection, only 12 participants' data (7 males and 5 females; mean age = 24.83 ± 2.33 years) were ultimately included in the analysis. All participants had no history of neurological or psychiatric conditions that could have affected the study outcomes. Prior to the experiment, each participant was informed in detail about the experimental procedures and provided written informed consent. The study protocol was approved by the Institutional Review Board (IRB) of Korea University (KUIRB-2023-0281-05).

### 2.2 taVNS application

**Figure 1:**
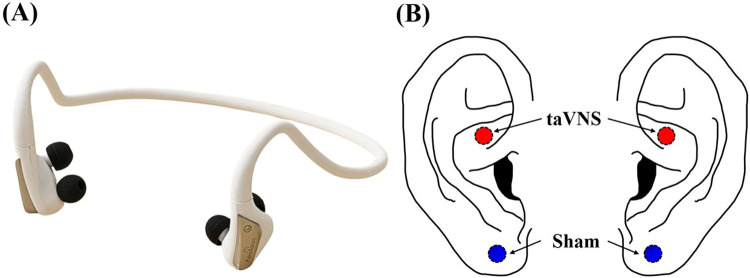
**(A)** taVNS device and **(B)** location of stimulation electrodes used in this study.

In this study, we employed a portable taVNS device equipped with two stimulation electrodes (Neurive, Co. Ltd., Gimhae, Republic of Korea) (Figure 1). Participants received either taVNS or sham stimulation in a randomized order, with a one-week interval between sessions. For taVNS, the electrodes were placed on the cymba conchae, where the vagus nerve is known to be present, whereas for sham stimulation, the electrodes were placed on the earlobe, which is considered devoid of vagus nerve innervation (Figure 1) (31, 42). During stimulation, the frequency was set to 30 Hz with a pulse width of 200 μs, and the protocol alternated 1-minute “on” periods of stimulation with 1-minute “off” (rest) periods (31, 43). Stimulation intensity was adjusted individually for each participant by gradually increasing from level 1 to level 10 until just below the participant's pain threshold (taVNS: 1.74 ± 0.06 mA, sham: 1.74 ± 0.04 mA). Our exclusion criterion was participants who reported pain even at the lowest intensity level (level 1), but no one met this condition.

### 2.3 Experimental protocol

**Figure 2:**
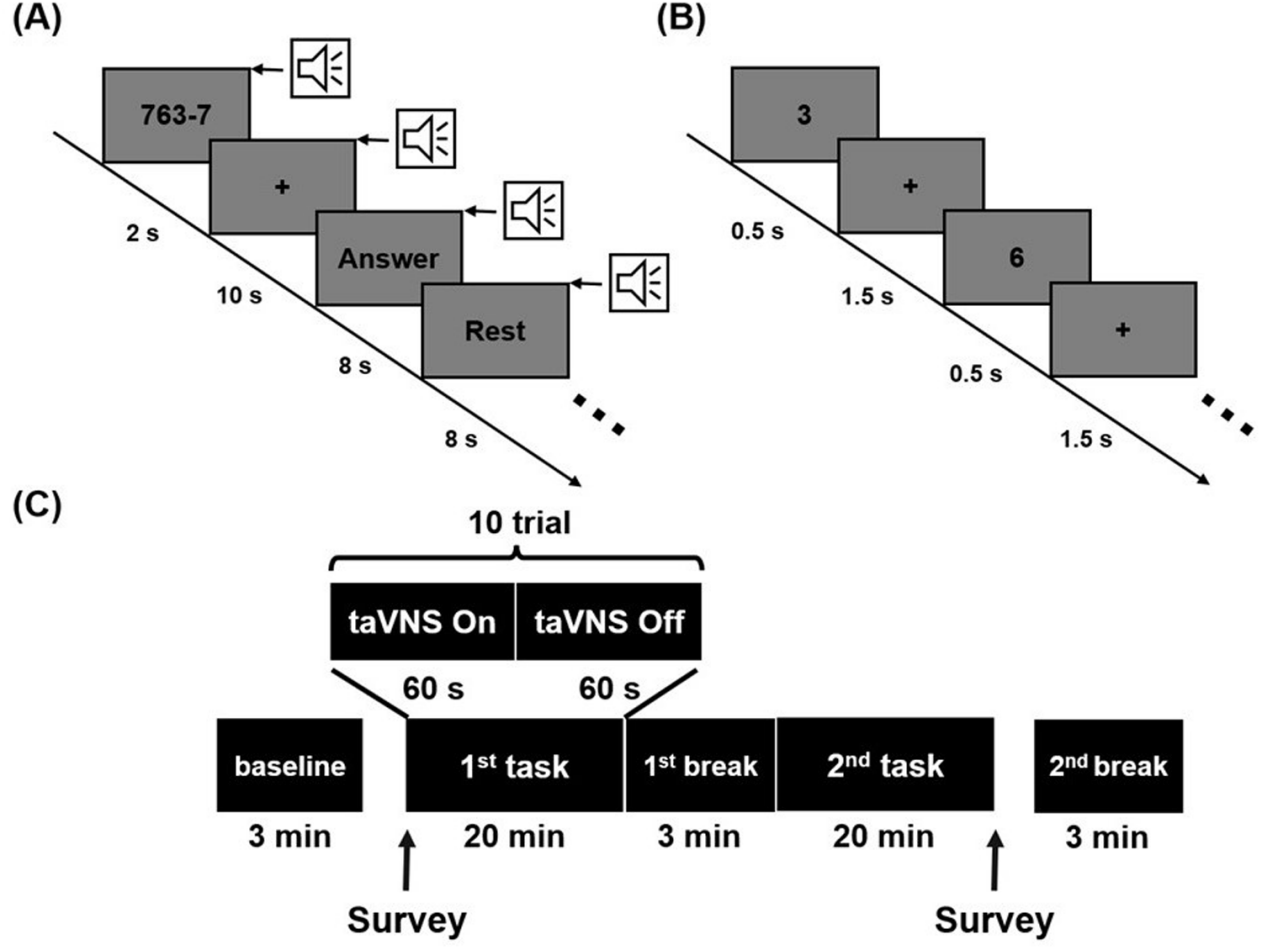
Experimental scheme illustrating **(A)** mental arithmetic task, **(B)** 2-back task and **(C)** the overall experimental paradigm. Stimulation was administered during each task, and resting-state EEG data were collected at baseline, the 1st break, and the 2nd break.

To investigate the effects of taVNS on stress, we utilized two mental stress-inducing tasks: the 2-back task and the mental arithmetic (MA) task (44–46). The 2-back task included a total of 600 trials. In each trial, a number from 1 to 9 was displayed at the center of the screen for 500 ms, followed by a fixation cross for 1,500 ms (Figure 2B). Participants were instructed to press the right arrow key if the presented number matched the one shown two trials earlier, and the left arrow key if it did not. One-third of the trials were target trials. The MA task consisted of 40 trials, each involving repeated subtraction of a single-digit number from a three-digit number (Figure 2A). In each trial, the three-digit and single-digit numbers were displayed for 2 s, followed by a 10-second period for participants to perform repeated subtraction and enter their answer. A 0.5 s beep was provided at the start of every trial to maintain participants' focus. If no answer was submitted within 8 s, the system automatically proceeded to a rest period, marking the trial as failed and ensuring at least 8 s of rest. The order of the two stress-inducing tasks was counter-balanced across participants, and the same task order was maintained for each participant during two experimental sessions conducted one week apart. Participants received either taVNS or sham stimulation in a counter-balanced order to minimize potential sequence effects between the two tasks and stimulation conditions.

Prior to any stimulation, a 3 min resting-state EEG was recorded to capture baseline conditions (“baseline” in Figure 2C). After each stress-inducing task, another 3 min resting-state EEG was recorded to assess the modulatory effects of taVNS (“1st break” and “2nd break” in Figure 2C). Although EEG data were also collected during the two tasks (“1st task” and “2nd task” in Figure 2C), these data were excluded from analysis due to significant electrical interference introduced by the taVNS stimulation. Note that previous studies have indicated that taVNS-induced changes in EEG activity can persist from several minutes (47) up to approximately 30 min (48) post-stimulation, suggesting that the resting-state EEG immediately recorded after stimulation could still reflect taVNS effects. In addition, participants completed a self-report survey before and after stimulation to measure subjective stress levels. This survey used a visual analog scale ranging from 1 to 10, where higher numbers indicate greater stress.

### 2.4 EEG recording and preprocessing

EEG data were recorded at a sampling rate of 1,000 Hz using 31 Ag/AgCl electrodes placed according to the international 10–20 system (Brain Products GmbH, Gilching, Germany). The reference and ground electrodes were positioned at FCz and AFz, respectively. Raw EEG signals were then downsampled to 200 Hz to reduce computational load. Common average referencing and 1–50 Hz band-pass filter were applied sequentially, and then independent component analysis (ICA) was performed to remove ocular and muscle artifacts. As baseline-based ICA evaluation was not applicable in our resting-state data (49), we instead applied a conservative component removal ratio, removing approximately one-third of the components on average (9.54 ± 0.89 out of 31), in line with previous research (50). The preprocessed EEG data were segmented into 5-second epochs, and any epoch exceeding ±75 μV in amplitude was excluded from power spectral density analysis (51). For the network analysis, segments identified as artifacts in any single channel were removed across all channels to maintain consistency in channel pairing. Consequently, artifact-free epochs were used for both PSD and network analyses. The total proportion of excluded epochs due to artifacts was approximately 0.45%.

### 2.5 PSD analysis

PSD was computed using short-time fast Fourier transform (FFT) with a 5 s window. PSD values were calculated for three frequency bands closely related to stress (52, 53): theta (4–7 Hz), alpha (8–13 Hz), and beta (14–30 Hz). To evaluate taVNS-driven stress modulation, the average PSD in each frequency band was computed specifically for the frontal electrodes (Fp1, Fp2, F3, F4, F7, F8), given their well-documented association with stress-related activity (54).

### 2.6 Network analysis

Network analysis was performed using imaginary coherence (iCoh) via the FieldTrip toolbox. iCoh was determined from spectral powers estimated by FFT with a single Hanning taper, ensuring a minimum of twenty cycles per frequency bin. While the PSD analysis focused on frontal channels closely linked to stress, the network analysis used all 31 electrodes to evaluate global brain network efficiency. Specifically, iCoh values were computed for each pair of the 31 electrodes across the theta, alpha, and beta frequency bands. From these iCoh matrices, we derived two global-level network indices—path length (PL) and clustering coefficient (CC)—using graph theoretical approaches. PL reflects the overall connectivity of the network, while CC indicates the clustering of neighboring nodes. All network computations were conducted using the Brain Connectivity Toolbox (BCT) in MATLAB.

### 2.7 Statistical analysis

As the data did not meet the assumptions of normality, non-parametric statistical methods were employed. To investigate taVNS-related changes in self-reported stress, Wilcoxon signed-rank tests were performed to compare pre- and post-stimulation survey scores. Additionally, the Friedman test was used to assess potential differences across baseline, the 1st break, and the 2nd break for both PSD and network indices. When significant effects were identified, *post-hoc* analyses were conducted using Wilcoxon signed-rank tests with false discovery rate (FDR) correction applied to account for multiple comparisons. Additionally, to complement the *p*-values and address concerns related to statistical power, we computed Wilcoxon effect sizes [*r* = |Z|/N; small: 0.1, medium: 0.3, large: 0.5] (55, 56).

## 3 Results

### 3.1 Stress level changes

**Figure 3:**
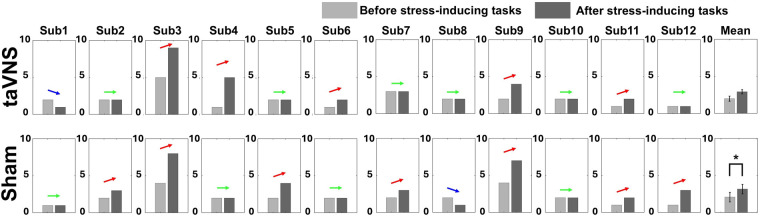
Changes in individual and averaged stress levels before and after two stress-inducing tasks for taVNS and sham conditions based on the self-report survey (*p < 0.05). Each bar graph represents the mean and standard error value.

Figure 3 illustrates both the individual and average changes in stress levels before and after stimulation for each condition, based on the self-report survey. In the sham condition, the mean stress level increased significantly after performing the task compared to before (*r* = 0.63, |Z| = 2.20, *p* < 0.05): seven participants reported increased stress levels, four showed no change, and one reported a decrease. In the taVNS condition, although the mean stress level also increased after the task, the change did not reach statistical significance (*r* = 0.52, |Z| = 1.80, *p* > 0.05): five participants reported increased stress, six reported no change, and one reported decreased stress. Note that there were no statistically significant differences in behavioral performance between taVNS and sham conditions for either task: MA accuracy (sham: 79.67 ± 3.11% vs. taVNS: 81.17 ± 3.72%) and average time required for one subtraction calculation (sham: 2.57 ± 0.22 s vs. taVNS: 2.61 ± 0.19 s); 2-back accuracy (sham: 83.36 ± 5.73% vs. taVNS: 83.26 ± 5.39%) and reaction time (sham: 658 ± 78 ms vs. taVNS: 648 ± 78 ms).

### 3.2 PSD modulation

**Figure 4:**
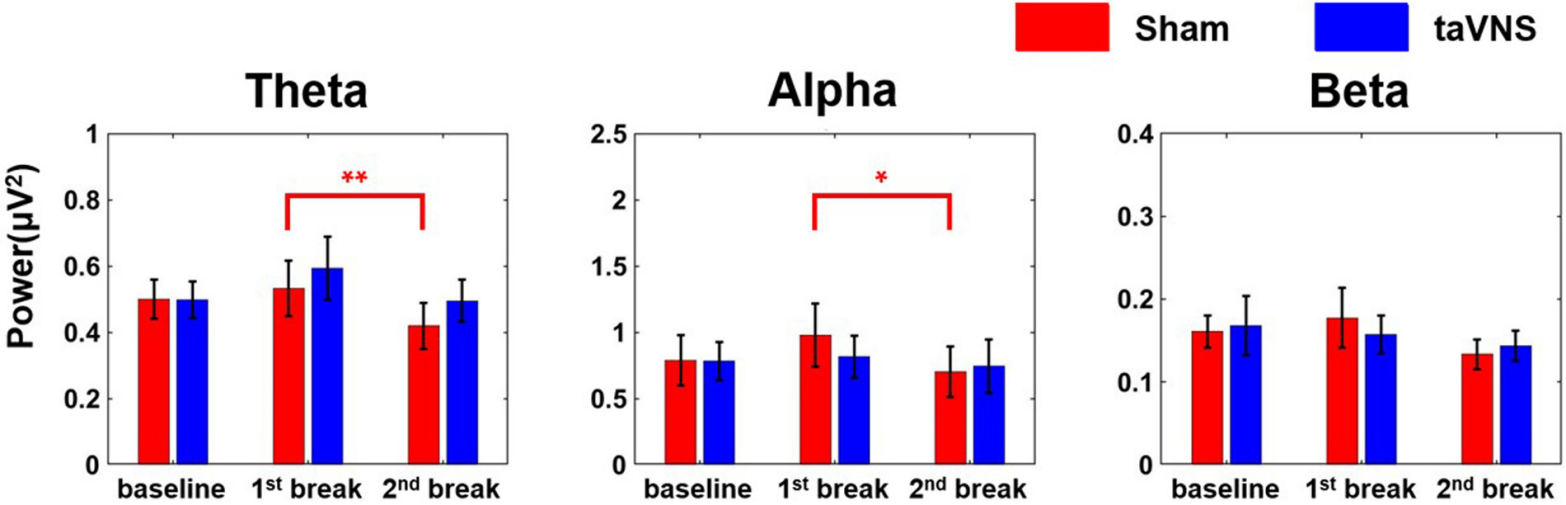
Changes in average PSD of each of the three frequency bands (theta, alpha, and beta) according to the stimulation condition in the frontal region (*p < 0.05, **p < 0.01). Each bar graph represents the mean and standard error value.

Figure 4 presents the average PSD changes for the three frequency bands (theta, alpha, and beta) in the frontal region, categorized by stimulation conditions. In the sham condition, theta and alpha PSDs decreased significantly from the 1st break to the 2nd break (theta: *r* = 0.86, |Z| = 2.98, *p* < 0.01, alpha: *r* = 0.79, |Z| = 2.75, *p* < 0.05), suggesting increased stress level, whereas no significant changes were observed in the taVNS condition. Neither the sham nor the taVNS condition showed significant changes in the beta band.

### 3.3 Network indices modulation

**Figure 5:**
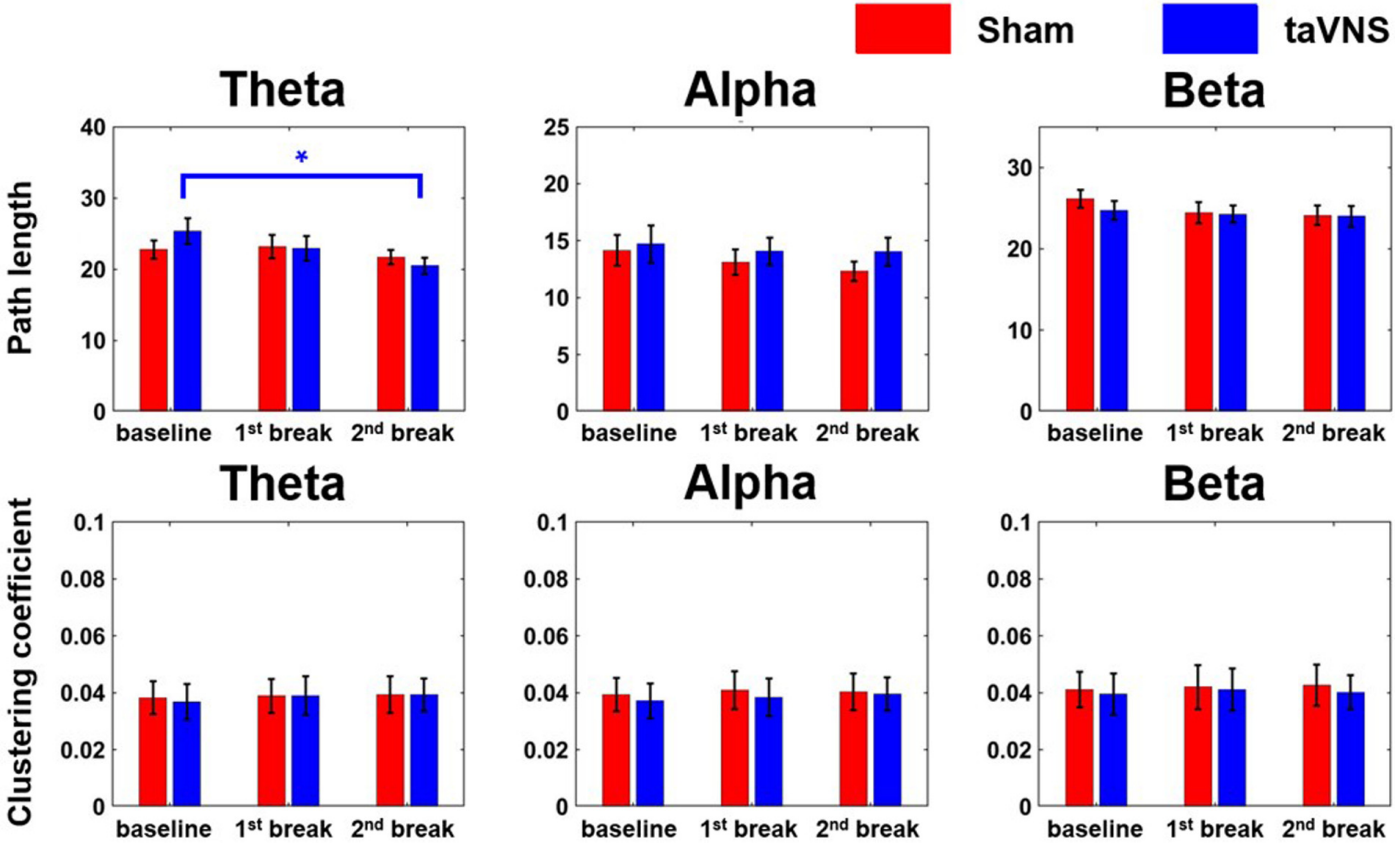
Changes in network indices (PL and CC) across the three frequency bands according to stimulation condition (*p < 0.05). Each bar graph represents the mean and standard error value.

Figure 5 shows the average changes in path length (PL) and clustering coefficient (CC) for the three frequency bands across the whole brain under each stimulation condition. In the taVNS condition, PL in the theta band decreased significantly at the 2nd break compared to baseline (*r* = 0.77, |Z| = 2.67, *p* < 0.05), indicating enhanced global network efficiency. By contrast, no significant changes were found in the sham condition. Regarding CC, neither condition showed significant changes across any of the frequency bands.

## 4 Discussion

In this study, we investigated the effects of taVNS on stress using self-report surveys and resting-state EEG recordings immediately following stimulation, capturing taVNS-induced neural changes. The survey results indicated a significant increase in stress levels in the sham condition after the task, whereas no statistically significant difference was observed in the taVNS condition. This finding was further supported by the PSD results, which showed a significant reduction in theta and alpha power at the 2nd break compared to the 1st break only in the sham condition, indicating a significant in stress level. Additionally, path length (PL) in the theta band was significantly reduced at the 2nd break compared to baseline under the taVNS condition, meaning enhanced network efficiency. These findings are consistent with previous studies demonstrating the stress-regulating effects of taVNS through ECG-based measures (37, 38, 57, 58), suggesting its potential efficacy in stress modulation. Therefore, our results suggest neurological evidence that taVNS can help modulate or suppress stress responses.

Through PSD analysis, we found that theta and alpha power decreased significantly at the 2nd break compared to the 1st break in the sham condition only. Previous studies have reported reductions in theta and alpha PSD alongside increases in beta PSD under elevated stress (52, 54, 59). While our results are consistent with these findings in terms of theta and alpha bands, they do not exhibit the same pattern in the beta band. One possible explanation is the difference in data used for analysis: previous studies showing elevated beta PSD during higher stress typically analyzed data recorded during stress-inducing tasks (54), whereas our study focused on resting-state EEG recorded before and after stress tasks. Another reason may be the neurophysiological trait that theta and alpha band activity is more prominently observed in resting-state conditions (60, 61). Consequently, the selective changes seen in these bands can provide reasonable evidence of taVNS effect on stress, given the nature of our EEG data collection.

Moreover, we observed a significant decrease in theta PL under the taVNS condition in the 2nd break compared to baseline. For instance, Shim et al. reported lower theta PL in healthy subjects compared to patients with PTSD (35), implying that stress might reduce overall brain network efficiency. Consistent with this, other studies have found that CC and small-worldness were lower in high-workload conditions than in low-workload conditions (36), suggesting that stressful situations negatively impact brain network function. Consequently, our finding of reduced PL under taVNS during a stressed condition implies that taVNS may enhance network efficiency and potentially help prevent further increase in stress.

An important observation is that the most prominent changes in PSD and network indices were identified at the 2nd break. Because participants underwent two distinct tasks over 40 min by the time of the 2nd break—compared to only one 20-minute task at the 1st break—the 2nd break induced higher stress levels. In the PSD results, significant reductions in theta and alpha bands were found in the sham condition at the 2nd break compared to the 1st break. These findings suggest that effects of taVNS on stress may become more evident under higher stress levels.

While our study presents neurophysiological evidence supporting the stress-regulating effects of taVNS, the participant pool was primarily composed of individuals in their early to mid-20 s, which may limit the generalizability of our findings. Furthermore, the limited number of participants remains a concern even though we presented effect sizes to enhance the reliability of our results. Future research should include a larger and more diverse sample to validate the broader applicability of taVNS and to further optimize stimulation parameters (e.g., duration, frequency, and pulse width) as well as the design of the sham condition (e.g., sham target location) used as a control group for maximizing its impact on stress regulation. In addition, although the stress-regulating effect of taVNS observed in this study may contribute to stress management in patients with stress-related disorders, our findings were derived from healthy participants only. Given that the effect sizes of significant EEG outcomes were large, it is reasonable to assume potential clinical relevance. However, clinical significance was not directly evaluated in this study. Therefore, further studies involving more diverse populations—including different age groups and individuals with clinical conditions—are needed to confirm the generalizability and establish the clinical applicability of taVNS.

## 5 Conclusion

This study investigated the effects of transcutaneous auricular vagus nerve stimulation (taVNS) on stress regulation using EEG measures and self-report surveys. The self-report data revealed a significant increase in stress levels under the sham condition, but not under taVNS. Additionally, both power spectral density (PSD) and network-based EEG indices indicated that taVNS may effectively mitigate rising stress levels and enhance brain connectivity. Although these findings underscore the potential of taVNS for stress modulation and neuromodulation, further research is needed to refine our understanding. Future studies should include more diverse participant groups and optimization of taVNS parameters. Such efforts will provide deeper insight into the mechanisms underlying stress-regulating effects of taVNS and enhance its potential clinical or practical applications.

## Data Availability

The raw data supporting the conclusions of this article will be made available by the authors, without undue reservation.
